# 861. Impact of COVID-19 Mitigation Measures on PrEP Care at a Safety Net Health System in Atlanta

**DOI:** 10.1093/ofid/ofab466.1056

**Published:** 2021-12-04

**Authors:** Valeria D Cantos, Kate Ferencsik, Jennifer Prevot, Kelly Reyna, Gabriela Georgial, Karla I Galaviz, Laris Niles-Carnes, Ziduo Zheng, Suprateek Kundu, Meredith Lora, Anandi N Sheth

**Affiliations:** 1 Emory University, Atlanta, Georgia; 2 Emory University School of Medicine, Atlanta, Georgia; 3 Indiana University School of Public Health, Bloomington, Indiana; 4 Grady Health System, Atlanta, Georgia

## Abstract

**Background:**

The Grady Health System pre-exposure prophylaxis (PrEP) program modified its care practices to accommodate COVID-19 mitigation measures. Changes enacted included: transition to telemedicine visits, medication mail delivery, and flexible timing of quarterly laboratory testing. These were implemented in March 2020 and remain in place presently. This study aimed to evaluate patients’ long-term acceptability of these modifications and to assess their impact on PrEP care.

**Methods:**

This was a cross-sectional study in a convenience sample of PrEP patients, ages 18 and older, at an urban clinic in Atlanta. They were invited to complete a survey between December 2020 and April 2021. The survey assessed the impact of mitigation measures on overall PrEP care, follow up visits, medication access, and ability to complete laboratory testing. It also evaluated the usability, quality, satisfaction, and concerns with telemedicine. Data were examined using median and interquartile ranges, and proportions.

**Results:**

Of 145 patients contacted, 61 completed the survey (median age 33 years, 72% Black, 75% cisgender men, 15% transgender women). Most participants did not report interruptions in their PrEP care (72%) or follow up visits (74%). Most found it easy to access medications (82%), as participants’ report of medication mail delivery usage increased from 57% (pre-pandemic) to 73% (in-pandemic period). Interruptions in completing quarterly labs were more frequently reported, as only 62% found this to be easy. Overall, 89% reported using telemedicine; telephone call was the most used method (78%). Telemedicine users’ ratings for quality, usability, and satisfaction of telemedicine was high (median score: 6/7) and nearly all users (97%) reported no concerns about its continued use for PrEP care. A few participants (5%) raised concerns about loss of telephone services due to financial issues, impacting their ability to complete telemedicine visits.

**Conclusion:**

PrEP care at an urban clinic was well- maintained despite COVID-19 mitigation measures. Telemedicine was found to be acceptable and usable by surveyed participants. Future research on widescale implementation of telemedicine for PrEP care is needed

**Disclosures:**

**All Authors**: No reported disclosures

